# Bis(4-acetyl­phen­yl) selenide

**DOI:** 10.1107/S1600536811009962

**Published:** 2011-03-23

**Authors:** Hazem Bouraoui, Ali Boudjada, Sofiane Bouacida, Youcef Mechehoud, Jean Meinnel

**Affiliations:** a Laboratoire de Cristallographie, Département de Physique, Université Mentouri-Constantine, 25000 Constantine, Algeria; b Unité de Recherche de Chimie de l’Environnement et Moléculaire Structurale, CHEMS, Université Mentouri-Constantine, 25000 Algeria; c Laboratoire VAREN, Département de Chimie, Faculté des Sciences Exactes, Université Mentouri-Constantine, 25000 Constantine, Algeria; d UMR 6226 CNRS–Université Rennes 1 ‘Sciences Chimiques de Rennes’, Equipe ‘Matière Condensée et Systèmes Electroactifs’, 263 Avenue du Général Leclerc, F-35042 Rennes, France

## Abstract

In the title compound, C_16_H_14_O_2_Se, the dihedral angle between the benzene rings is 87.08 (11)°. In the crystal, mol­ecules are linked into layers parallel to the *bc* plane by inter­molecular C—H⋯O hydrogen bonds.

## Related literature

For the synthesis of the title compound, see: Henry (1943 ▶). For biological properties and applications of organoselenide compounds, see: Clement *et al.* (1997 ▶); Anderson *et al.* (1996 ▶); Abdel-Hafez (2008 ▶); Woods *et al.* (1993 ▶); Hellberg *et al.* (1997 ▶). For a description of the Cambridge Structural Database, see: Allen (2002 ▶).
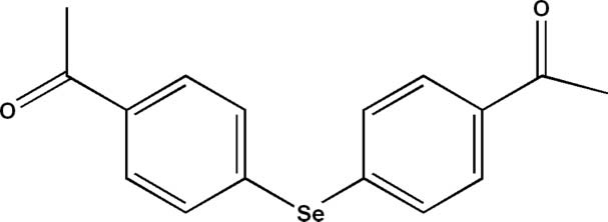


## Experimental

### 

#### Crystal data

C_16_H_14_O_2_Se
*M* *_r_* = 317.23
Monoclinic, 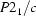

*a* = 14.9290 (7) Å
*b* = 7.7223 (3) Å
*c* = 13.8345 (6) Å
β = 115.993 (2)°
*V* = 1433.60 (11) Å^3^
*Z* = 4
Mo *K*α radiation
μ = 2.61 mm^−1^
*T* = 295 K
0.14 × 0.07 × 0.05 mm

#### Data collection

Nonius KappaCCD diffractometer
6274 measured reflections
3272 independent reflections
1904 reflections with *I* > 2σ(*I*)
*R*_int_ = 0.027

#### Refinement

*R*[*F* ^2^ > 2σ(*F* ^2^)] = 0.041
*wR*(*F* ^2^) = 0.112
*S* = 1.04
3272 reflections
174 parameters
H-atom parameters constrained
Δρ_max_ = 0.48 e Å^−3^
Δρ_min_ = −0.59 e Å^−3^

### 

Data collection: *KappaCCD Reference Manual* (Nonius, 1998 ▶); cell refinement: *SCALEPACK* (Otwinowski & Minor, 1997 ▶); data reduction: *SCALEPACK* and *DENZO* (Otwinowski & Minor, 1997 ▶); program(s) used to solve structure: *SIR2002* (Burla *et al.*, 2003 ▶); program(s) used to refine structure: *SHELXL97* (Sheldrick, 2008 ▶); molecular graphics: *ORTEP-3* (Farrugia, 1997 ▶) and *DIAMOND* (Brandenburg & Berndt, 2001 ▶); software used to prepare material for publication: *WinGX* (Farrugia, 1999 ▶).

## Supplementary Material

Crystal structure: contains datablocks global, I. DOI: 10.1107/S1600536811009962/rz2569sup1.cif

Structure factors: contains datablocks I. DOI: 10.1107/S1600536811009962/rz2569Isup2.hkl

Additional supplementary materials:  crystallographic information; 3D view; checkCIF report

## Figures and Tables

**Table 1:** Hydrogen-bond geometry (Å, °)

*D*—H⋯*A*	*D*—H	H⋯*A*	*D*⋯*A*	*D*—H⋯*A*
C2—H2⋯O2^i^	0.93	2.47	3.272 (5)	145
C12—H12⋯O1^ii^	0.93	2.53	3.317 (4)	143

Symmetry codes: (i) 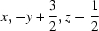; (ii) 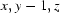.

**Fig. 1.**
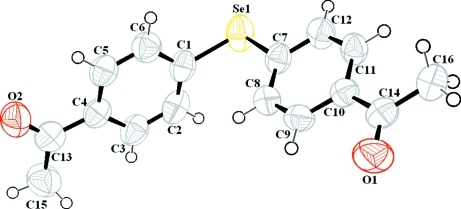
The molecular structure of the title compound with displacement ellipsoids drawn at the 50% probability level.

**Fig. 2.**
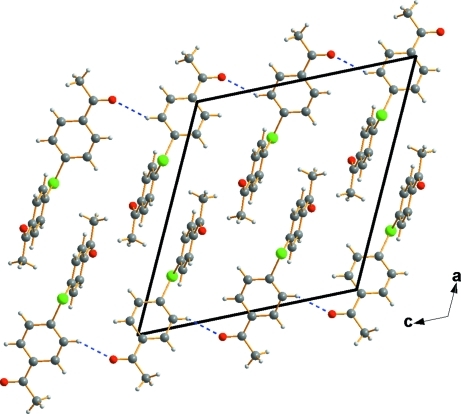
Packing diagram of the title compound viewed down the b axis showing the chains parallel to the c axis formed by hydrogen bonds (dashed line).

**Fig. 3.**
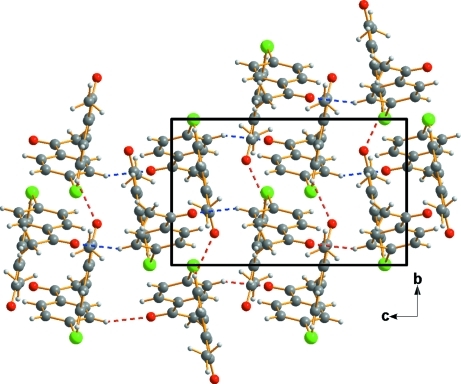
Crystal packing of the title compound viewed down the a axis showing a layer parallel to the bc plane. Hhydrogen bonds are shown as dashed lines.

## supplementary crystallographic information

### Comment

Organoselenides and derivatives are of considerable interest in academia as anti-cancer (Clement *et al.*, 1997), anti-oxydant (Anderson *et al.*, 1996), anti-inflammatory and antiallergic agents (Abdel-Hafez, 2008), and in industry because of their wide involvement as key intermediates for the synthesis of pharmaceuticals (Woods *et al.*, 1993), perfumes, fine chemicals and polymers (Hellberg *et al.*, 1997). In the framework of our ongoing program related to the synthesis and pharmaceutical evaluation of new organoselenide derivatives, we report here the synthesis and crystal structure of the title compound.

In the molecule of the title compound (Fig. 1), the selenium atom is linked to two phenyl acetyl groups. All bond distances and angles are within the ranges of accepted values (CSD; Allen, 2002). The molecule is not planar, as can be seen from the dihedral angle of 87.08 (11)° between the planes of the two benzene rings. In the crystal structure, molecules are linked into chains running parallel to the *c* axis by intermolecular C2—H2···O2 hydrogen interactions (Fig. 2, Table 1). The chains are further connected by C12—H12···O1 hydrogen bonds to form layers parallel to the *bc* plane (Fig. 3).

### Experimental

The title compound was prepared according to a literature method (Henry, 1943). Methyl acyl chloride (2.4 mmol) and anhydrous aluminium chloride (3.0 mmol) were dissolved in dry methylene chloride (4 ml). The reaction mixture was cooled at 0–5 °C, protected from atmospheric moisture, and stirred continuously from 15 min. A solution of diphenyl selenide (1 mmol) in methylene chloride (0.5 ml) was then added dropwise over a period of 5 min. The reaction mixture was allowed to reach room temperature gradually and stirred at this temperature overnight. The solution was then washed with ice water-HCl and extracted with methylene chloride. The organic layer was separated and dried over Na_2_SO_4_. Removal of the solvent afforded the crude title product which was recrystallized from CH_3_OH. Some crystals suitable for X-ray diffraction analysis were carefully isolated under polarizing microscope.

### Refinement

All H atoms were localized in a Fourier difference map and introduced in calculated positions as riding on their parent C atoms, with C_aryl_—H = 0.93 Å, C_methyl_—H = 0.96 Å, and with *U*_iso_(H) = 1.5*U*_eq_(C_methyl_) or *U*_iso_(H) = 1.2*U*_eq_(C_aryl_).

### Crystal data

**Table:**

C_16_H_14_O_2_Se	*F*(000) = 640
*M**_r_* = 317.23	*D*_x_ = 1.47 Mg m^−^^3^
Monoclinic, *P*2_1_/*c*	Mo *K*α radiation, λ = 0.71073 Å
*a* = 14.9290 (7) Å	Cell parameters from 9479 reflections
*b* = 7.7223 (3) Å	θ = 2.9–27.5°
*c* = 13.8345 (6) Å	µ = 2.61 mm^−^^1^
β = 115.993 (2)°	*T* = 295 K
*V* = 1433.60 (11) Å^3^	Needle, white
*Z* = 4	0.14 × 0.07 × 0.05 mm

### Data collection

**Table:**

Nonius KappaCCD diffractometer	*R*_int_ = 0.027
graphite	θ_max_ = 27.5°, θ_min_ = 3.0°
CCD rotation images, thick slices scans	*h* = −19→19
6274 measured reflections	*k* = −9→10
3272 independent reflections	*l* = −17→17
1904 reflections with *I* > 2σ(*I*)	

### Refinement

**Table:**

Refinement on *F*^2^	Primary atom site location: structure-invariant direct methods
Least-squares matrix: full	Secondary atom site location: difference Fourier map
*R*[*F*^2^ > 2σ(*F*^2^)] = 0.041	Hydrogen site location: inferred from neighbouring sites
*wR*(*F*^2^) = 0.112	H-atom parameters constrained
*S* = 1.04	*w* = 1/[σ^2^(*F*_o_^2^) + (0.0407*P*)^2^ + 0.5483*P*] where *P* = (*F*_o_^2^ + 2*F*_c_^2^)/3
3272 reflections	(Δ/σ)_max_ < 0.001
174 parameters	Δρ_max_ = 0.48 e Å^−^^3^
0 restraints	Δρ_min_ = −0.58 e Å^−^^3^

### Special details

**Table:**

Geometry. All e.s.d.'s (except the e.s.d. in the dihedral angle between two l.s. planes) are estimated using the full covariance matrix. The cell e.s.d.'s are taken into account individually in the estimation of e.s.d.'s in distances, angles and torsion angles; correlations between e.s.d.'s in cell parameters are only used when they are defined by crystal symmetry. An approximate (isotropic) treatment of cell e.s.d.'s is used for estimating e.s.d.'s involving l.s. planes.
Refinement. Refinement of *F*^2^ against ALL reflections. The weighted *R*-factor *wR* and goodness of fit *S* are based on *F*^2^, conventional *R*-factors *R* are based on *F*, with *F* set to zero for negative *F*^2^. The threshold expression of *F*^2^ > σ(*F*^2^) is used only for calculating *R*-factors(gt) *etc*. and is not relevant to the choice of reflections for refinement. *R*-factors based on *F*^2^ are statistically about twice as large as those based on *F*, and *R*- factors based on ALL data will be even larger.

### Fractional atomic coordinates and isotropic or equivalent isotropic displacement parameters (Å^2^)

**Table:**

	*x*	*y*	*z*	*U*_iso_*/*U*_eq_	
C1	0.1305 (3)	0.6107 (4)	0.4328 (3)	0.0693 (8)	
C2	0.0389 (3)	0.6402 (5)	0.3498 (3)	0.0807 (10)	
H2	0.0275	0.6113	0.2801	0.097*	
C3	−0.0369 (3)	0.7122 (5)	0.3678 (3)	0.0758 (9)	
H3	−0.0985	0.7327	0.3101	0.091*	
C4	−0.0222 (2)	0.7546 (4)	0.4719 (2)	0.0631 (8)	
C5	0.0705 (3)	0.7225 (4)	0.5546 (3)	0.0699 (8)	
H5	0.0820	0.7490	0.6247	0.084*	
C6	0.1463 (3)	0.6524 (4)	0.5365 (3)	0.0727 (9)	
H6	0.2083	0.6330	0.5939	0.087*	
C7	0.2962 (2)	0.7077 (4)	0.3840 (2)	0.0610 (7)	
C8	0.2529 (2)	0.8705 (4)	0.3634 (2)	0.0643 (8)	
H8	0.1896	0.8864	0.3594	0.077*	
C9	0.3038 (2)	1.0081 (4)	0.3489 (2)	0.0614 (7)	
H9	0.2743	1.1170	0.3352	0.074*	
C10	0.3987 (2)	0.9885 (4)	0.3543 (2)	0.0550 (7)	
C11	0.4414 (2)	0.8249 (4)	0.3750 (2)	0.0618 (8)	
H11	0.5049	0.8091	0.3793	0.074*	
C12	0.3904 (2)	0.6849 (4)	0.3891 (3)	0.0683 (8)	
H12	0.4194	0.5755	0.4021	0.082*	
C13	−0.1026 (3)	0.8262 (4)	0.4962 (3)	0.0715 (9)	
C14	0.4524 (2)	1.1412 (4)	0.3393 (2)	0.0613 (8)	
C15	−0.2035 (3)	0.8594 (6)	0.4069 (3)	0.0998 (13)	
H15A	−0.2445	0.9155	0.4350	0.150*	
H15B	−0.2335	0.7515	0.3743	0.150*	
H15C	−0.1974	0.9326	0.3540	0.150*	
C16	0.5559 (2)	1.1204 (5)	0.3517 (3)	0.0722 (9)	
H16A	0.5821	1.2315	0.3462	0.108*	
H16B	0.5557	1.0456	0.2962	0.108*	
H16C	0.5968	1.0707	0.4208	0.108*	
O1	0.41088 (19)	1.2823 (3)	0.3175 (2)	0.0912 (8)	
O2	−0.08802 (19)	0.8541 (4)	0.5879 (2)	0.0940 (8)	
Se1	0.23415 (3)	0.50420 (5)	0.40667 (4)	0.08634 (18)	

### Atomic displacement parameters (Å^2^)

**Table:**

	*U*^11^	*U*^22^	*U*^33^	*U*^12^	*U*^13^	*U*^23^
C1	0.078 (2)	0.068 (2)	0.074 (2)	−0.0169 (17)	0.0436 (19)	−0.0041 (16)
C2	0.092 (3)	0.094 (3)	0.064 (2)	−0.023 (2)	0.041 (2)	−0.0082 (18)
C3	0.070 (2)	0.095 (3)	0.0593 (19)	−0.0153 (19)	0.0254 (17)	−0.0016 (17)
C4	0.069 (2)	0.0613 (18)	0.0599 (19)	−0.0142 (15)	0.0291 (17)	0.0000 (14)
C5	0.076 (2)	0.077 (2)	0.0569 (18)	−0.0084 (18)	0.0297 (17)	−0.0080 (16)
C6	0.071 (2)	0.078 (2)	0.070 (2)	−0.0039 (17)	0.0309 (18)	−0.0003 (17)
C7	0.075 (2)	0.0568 (17)	0.0604 (17)	−0.0054 (15)	0.0377 (15)	−0.0072 (14)
C8	0.0614 (19)	0.068 (2)	0.0678 (19)	0.0008 (15)	0.0326 (16)	0.0010 (15)
C9	0.0596 (18)	0.0568 (17)	0.0649 (18)	0.0086 (15)	0.0245 (15)	0.0081 (15)
C10	0.0651 (18)	0.0518 (16)	0.0498 (15)	0.0013 (14)	0.0268 (13)	−0.0029 (13)
C11	0.070 (2)	0.0547 (17)	0.0726 (19)	0.0023 (15)	0.0428 (17)	−0.0032 (14)
C12	0.088 (2)	0.0492 (16)	0.084 (2)	0.0072 (15)	0.0521 (19)	−0.0029 (15)
C13	0.074 (2)	0.066 (2)	0.073 (2)	−0.0098 (16)	0.0311 (18)	−0.0018 (17)
C14	0.071 (2)	0.0548 (18)	0.0572 (17)	−0.0027 (15)	0.0275 (16)	0.0018 (14)
C15	0.073 (3)	0.119 (4)	0.097 (3)	0.007 (2)	0.028 (2)	−0.001 (2)
C16	0.079 (2)	0.075 (2)	0.072 (2)	−0.0066 (18)	0.0422 (18)	0.0016 (17)
O1	0.0897 (17)	0.0572 (14)	0.126 (2)	0.0055 (13)	0.0468 (16)	0.0179 (14)
O2	0.0921 (18)	0.116 (2)	0.0801 (17)	0.0134 (15)	0.0433 (14)	−0.0069 (15)
Se1	0.1093 (3)	0.0598 (2)	0.1188 (4)	−0.0126 (2)	0.0767 (3)	−0.0080 (2)

### Geometric parameters (Å, °)

**Table:**

C13—O2	1.209 (4)	C2—H2	0.9300
C13—C4	1.486 (4)	C3—C4	1.397 (4)
C13—C15	1.494 (5)	C3—H3	0.9300
C14—O1	1.224 (4)	C4—C5	1.378 (4)
C14—C16	1.488 (4)	C5—C6	1.373 (4)
C14—C10	1.490 (4)	C5—H5	0.9300
C15—H15A	0.9600	C6—H6	0.9300
C15—H15B	0.9600	C7—C8	1.385 (4)
C15—H15C	0.9600	C7—C12	1.389 (4)
C16—H16A	0.9600	C8—C9	1.371 (4)
C16—H16B	0.9600	C8—H8	0.9300
C16—H16C	0.9600	C9—C10	1.394 (4)
Se1—C7	1.918 (3)	C9—H9	0.9300
Se1—C1	1.921 (3)	C10—C11	1.387 (4)
C1—C2	1.366 (5)	C11—C12	1.385 (4)
C1—C6	1.386 (4)	C11—H11	0.9300
C2—C3	1.378 (5)	C12—H12	0.9300
			
O2—C13—C4	120.8 (3)	C5—C4—C3	117.4 (3)
O2—C13—C15	119.3 (3)	C5—C4—C13	119.7 (3)
C4—C13—C15	119.9 (3)	C3—C4—C13	122.9 (3)
O1—C14—C16	120.8 (3)	C6—C5—C4	121.9 (3)
O1—C14—C10	119.6 (3)	C6—C5—H5	119.1
C16—C14—C10	119.6 (3)	C4—C5—H5	119.1
C13—C15—H15A	109.5	C5—C6—C1	120.0 (3)
C13—C15—H15B	109.5	C5—C6—H6	120.0
H15A—C15—H15B	109.5	C1—C6—H6	120.0
C13—C15—H15C	109.5	C8—C7—C12	119.7 (3)
H15A—C15—H15C	109.5	C8—C7—Se1	124.2 (2)
H15B—C15—H15C	109.5	C12—C7—Se1	116.0 (2)
C14—C16—H16A	109.5	C9—C8—C7	119.7 (3)
C14—C16—H16B	109.5	C9—C8—H8	120.1
H16A—C16—H16B	109.5	C7—C8—H8	120.1
C14—C16—H16C	109.5	C8—C9—C10	121.5 (3)
H16A—C16—H16C	109.5	C8—C9—H9	119.2
H16B—C16—H16C	109.5	C10—C9—H9	119.2
C7—Se1—C1	99.58 (13)	C11—C10—C9	118.3 (3)
C2—C1—C6	119.0 (3)	C11—C10—C14	121.5 (3)
C2—C1—Se1	120.3 (3)	C9—C10—C14	120.2 (3)
C6—C1—Se1	120.6 (3)	C12—C11—C10	120.6 (3)
C1—C2—C3	120.9 (3)	C12—C11—H11	119.7
C1—C2—H2	119.5	C10—C11—H11	119.7
C3—C2—H2	119.5	C11—C12—C7	120.1 (3)
C2—C3—C4	120.7 (3)	C11—C12—H12	120.0
C2—C3—H3	119.6	C7—C12—H12	120.0
C4—C3—H3	119.6		
			
C7—Se1—C1—C2	91.3 (3)	C1—Se1—C7—C8	−15.7 (3)
C7—Se1—C1—C6	−91.3 (3)	C1—Se1—C7—C12	164.3 (2)
C6—C1—C2—C3	0.7 (5)	C12—C7—C8—C9	−0.4 (5)
Se1—C1—C2—C3	178.2 (3)	Se1—C7—C8—C9	179.6 (2)
C1—C2—C3—C4	−0.9 (5)	C7—C8—C9—C10	0.0 (5)
C2—C3—C4—C5	0.3 (5)	C8—C9—C10—C11	0.0 (4)
C2—C3—C4—C13	−177.6 (3)	C8—C9—C10—C14	−179.4 (3)
O2—C13—C4—C5	−1.2 (5)	O1—C14—C10—C11	177.4 (3)
C15—C13—C4—C5	−179.3 (3)	C16—C14—C10—C11	−2.9 (4)
O2—C13—C4—C3	176.7 (3)	O1—C14—C10—C9	−3.3 (4)
C15—C13—C4—C3	−1.5 (5)	C16—C14—C10—C9	176.4 (3)
C3—C4—C5—C6	0.3 (5)	C9—C10—C11—C12	0.4 (4)
C13—C4—C5—C6	178.3 (3)	C14—C10—C11—C12	179.7 (3)
C4—C5—C6—C1	−0.5 (5)	C10—C11—C12—C7	−0.7 (5)
C2—C1—C6—C5	−0.1 (5)	C8—C7—C12—C11	0.7 (5)
Se1—C1—C6—C5	−177.5 (3)	Se1—C7—C12—C11	−179.3 (2)

### Hydrogen-bond geometry (Å, °)

**Table:**

*D*—H···*A*	*D*—H	H···*A*	*D*···*A*	*D*—H···*A*
C2—H2···O2^i^	0.93	2.47	3.272 (5)	145
C12—H12···O1^ii^	0.93	2.53	3.317 (4)	143

Symmetry codes: (i) *x*, −*y*+3/2, *z*−1/2; (ii) *x*, *y*−1, *z*.
